# Mobile-assisted deep learning framework for identification of insect pests and diseases of maize from field images

**DOI:** 10.3389/fpls.2026.1803005

**Published:** 2026-04-27

**Authors:** Niranjan Singh, Meenakshi Malik, Mukesh Kumar Khokhar, Anoop Kumar, Manoj Choudhary, Mohit Kumar, Niranjan Nayak, Ajay Kumar Singh

**Affiliations:** ICAR- National Research Institute for Integrated Pest Management, New Delhi, India

**Keywords:** deep learning, disease detection, maize, MobileViT, YOLO

## Abstract

Maize (*Zea mays L.*) production is severely affected by diseases and insect pests, leading to significant yield losses when timely diagnosis and management interventions are not implemented. Although automated image-based diagnostic systems have shown promising results, most existing studies address diseases or pests independently, rely on controlled datasets, and offer limited robustness under real field conditions. To address these limitations, this study proposes a unified deep learning–based framework for integrated identification of maize diseases and insect pests under natural field environments by combining object detection and image classification within a mobile-assisted diagnostic system. Four economically important diseases and insect pests were investigated: Maydis Leaf Blight (MLB), Turcicum Leaf Blight (TLB), Common Rust, and Fall Armyworm (FAW). MLB and TLB were addressed using YOLO-based object detection architectures, while Common Rust and FAW were treated as image-level classification tasks using lightweight deep learning models optimised for mobile inference. A self-collected dataset comprising 10,343 images across four classes was acquired under real field conditions to capture variability in background complexity, illumination, phenological stages, and symptom expression. Experimental results on an independent test set comprising original images demonstrate that MobileViT achieved the highest classification accuracy (99%) for image-level disease and pest recognition, whereas YOLOv11n outperformed other detection models, achieving the best performance for MLB and TLB lesion detection with mAP@0.5 of 0.875. Grad-CAM–based visual explanation analysis confirmed that the classification models focused on disease lesions and pest-infested regions, supporting interpretability. The framework was successfully deployed via a mobile application, enabling image acquisition, automated validation, diagnosis, and the generation of management recommendations. The results highlight the accuracy, robustness, and operational feasibility of the proposed system for in-field diagnosis of maize diseases and insect pests, supporting early detection and sustainable crop protection.

## 1 Introduction

Maize (*Zea mays L.*) is one of the most widely cultivated cereal crops worldwide and serves as a staple source of food, feed, and industrial raw material. Owing to its high productivity and broad adaptability, maize plays a critical role in global food security, particularly in developing countries such as India (Padhan et al., 2024). However, maize production is severely constrained by biotic stresses, including diseases and insect pests, which affect the crop at multiple growth stages and cause substantial yield losses (Savary et al., 2019).

Among the most damaging diseases of maize are MLB (*Bipolaris maydis*), Urvularia leaf spot (*Curvularia lunata*), Banded leaf and sheath blight (*Rhizoctonia solani f.* sp. *sasakii*), Rust (*Puccinia sorghi*), Brown spot (*Physoderma zeae-maydis*), and TLB (*Exserohilum turcicum*) (Pattanayak et al., 2025). In addition, insect pests such as FAW (*Spodoptera frugiperda*) and stem borer (*Chilo partellus*) have emerged as major threats in recent years (Prasanna et al., 2022). Under favourable environmental conditions, these stresses can result in yield losses of up to 30–35%, particularly when detection and management interventions are not carried out in a timely manner (Goergen et al., 2016; Prasanna et al., 2018). Timely and accurate identification of both diseases and insect pests is therefore essential for effective crop protection and sustainable maize production.

In current agricultural practice, disease and pest identification relies largely on visual inspection by farmers or extension personnel. Such assessments are inherently subjective, labour-intensive, and often unreliable, especially for smallholder farmers with limited technical training. Although expert consultation improves diagnostic accuracy, it is frequently inaccessible or unaffordable in rural regions (Ouppaphan, 2017). Consequently, farmers often depend on pesticide dealers for advisory support, leading to excessive or inappropriate chemical usage. This practice not only increases production costs but also poses serious risks to environmental sustainability, human health, beneficial insects, and pollinator populations (Pretty and Bharucha, 2015; Damalas and Koutroubas, 2016).

Automated image-based identification systems have emerged as a promising alternative for improving the accuracy, timeliness, and scalability of maize disease and pest monitoring. Early studies employing conventional machine learning techniques demonstrated potential for maize disease recognition; however, their reliance on handcrafted feature extraction limited robustness and generalization under real field conditions (Barbedo, 2018; Panigrahi et al., 2020; Kundu et al., 2022). These limitations became particularly evident in complex agricultural environments characterised by variable illumination, background clutter, and heterogeneous symptom expression.

Recent advances in deep learning, particularly Convolutional Neural Networks (CNNs), have substantially improved performance in image classification and object detection tasks (Dagar et al., 2024; Kumar et al., 2024). Since the introduction of AlexNet (Krizhevsky et al., 2012), CNN-based methods have been widely adopted in plant pathology, with several studies demonstrating their effectiveness for maize disease identification under field conditions (DeChant et al., 2017; Ahila Priyadharshini et al., 2019; Dhaka et al., 2021). Furthermore, the development of lightweight CNN architectures has enabled deployment on mobile and edge devices, facilitating real-time decision support for farmers (Sandler et al., 2018; Howard et al., 2019; Kumar et al., 2025).

Despite these advances, existing research exhibits several important limitations. Most studies address either disease classification or pest identification as isolated problems, rather than considering the broader spectrum of maize biotic stresses encountered in practice. Many approaches rely exclusively on image-level classification, which is insufficient for diseases characterized by localised lesion patterns that require precise spatial localisation (Ferentinos, 2018). In addition, a large proportion of existing work is based on publicly available or previously collected datasets (Craze et al., 2022; Pandey et al., 2023; Srivastav et al., 2024), often acquired under controlled conditions, limiting their applicability to real-world field environments. Limited attention has also been given to unified frameworks that integrate both detection and classification while explicitly considering deployment constraints on mobile platforms. YOLO-based object detection frameworks have become increasingly preferred for plant disease detection due to their ability to perform accurate and real-time localisation of disease symptoms under complex field conditions. Unlike conventional image classification approaches that only identify the presence of disease at the image-level, YOLO models enable precise detection of lesion regions, allowing more informative diagnosis and interpretation of plant health status. Several studies have demonstrated the effectiveness of YOLO architectures for detecting plant disease symptoms and leaf lesions across different crops (Mathew and Mahesh, 2022; Alhwaiti et al., 2025; Kaur et al., 2025). In addition, recent developments in YOLO-based frameworks for agricultural monitoring have further highlighted the advantages of these models for crop phenotyping and automated field analysis (Li et al., 2025; You et al., 2025; Zhao et al., 2025). YOLO-based models offer advantages such as fast inference speed, efficient feature extraction, and the ability to handle complex backgrounds commonly encountered in agricultural environments. Consequently, YOLO variants have emerged as a widely adopted solution for plant disease and pest detection tasks in precision agriculture. However, most existing studies focus primarily on single-task detection scenarios and do not address the combined challenge of detecting spatially localised disease lesions while simultaneously identifying pest or disease symptoms that appear in a more diffuse manner.

To address these gaps, this study proposes a comprehensive deep learning–based framework for maize disease and insect pest identification under natural field conditions. The framework integrates object detection and image classification to address different symptom characteristics. Diseases characterized by localised lesion patterns, MLB and TLB, are formulated as object detection problems using YOLO-based architectures. In this study, lightweight variants of YOLO models (YOLOv5n, YOLOv8n, and YOLOv11n) were systematically evaluated to determine their suitability for lesion detection in maize leaves. These models were selected due to their efficient architecture, high detection accuracy, and real-time inference capability, making them particularly suitable for deployment in resource-constrained environments such as mobile-assisted agricultural systems (Jocher et al., 2023, 2024). In contrast, Common Rust and FAW damage are addressed as image-level classification tasks using mobile-friendly deep learning architectures, including MobileNetV4 and MobileViT (Howard et al., 2019; Mehta and Rastegari, 2021), selected for their efficiency and strong performance in real-world image classification tasks.

A balanced dataset comprising 10,343 field images was collected under natural agricultural conditions and augmented to capture realistic variability in background complexity, illumination conditions, crop growth stages, and symptom severity across diverse agro-ecological environments. The proposed framework emphasizes practical applicability through integration with a mobile application supported by a backend inference system, enabling both disease and pest identification along with advisory support. By combining lesion-level object detection for spatially localised diseases with image-level classification for diffuse pest and disease symptoms, and by systematically comparing multiple lightweight YOLO detection variants alongside efficient classification architectures, this study provides a reproducible benchmark and a practical solution for precision maize health monitoring under real field conditions.

## 2 Materials and methods

### 2.1 Study design and workflow

This research presents a deep learning–based framework for automated detection and classification of maize diseases and insect pests using leaf imagery. The dataset comprises four maize disease and insect pest classes: MLB, TLB, Common Rust, and FAW. MLB and TLB were formulated as object detection problems because symptoms appear as multiple, spatially distinct lesions requiring localization. In contrast, Common Rust and FAW were addressed using image-level classification, as the dense, overlapping pustules and feeding damage made object-level annotation difficult and inconsistent in field-acquired images.

The overall workflow consists of dataset preparation, annotation, data augmentation, model training, validation, and quantitative evaluation. Separate model architectures were selected for detection and classification tasks to ensure methodological suitability and performance reliability.

### 2.2 Dataset description

**Figure 1:**
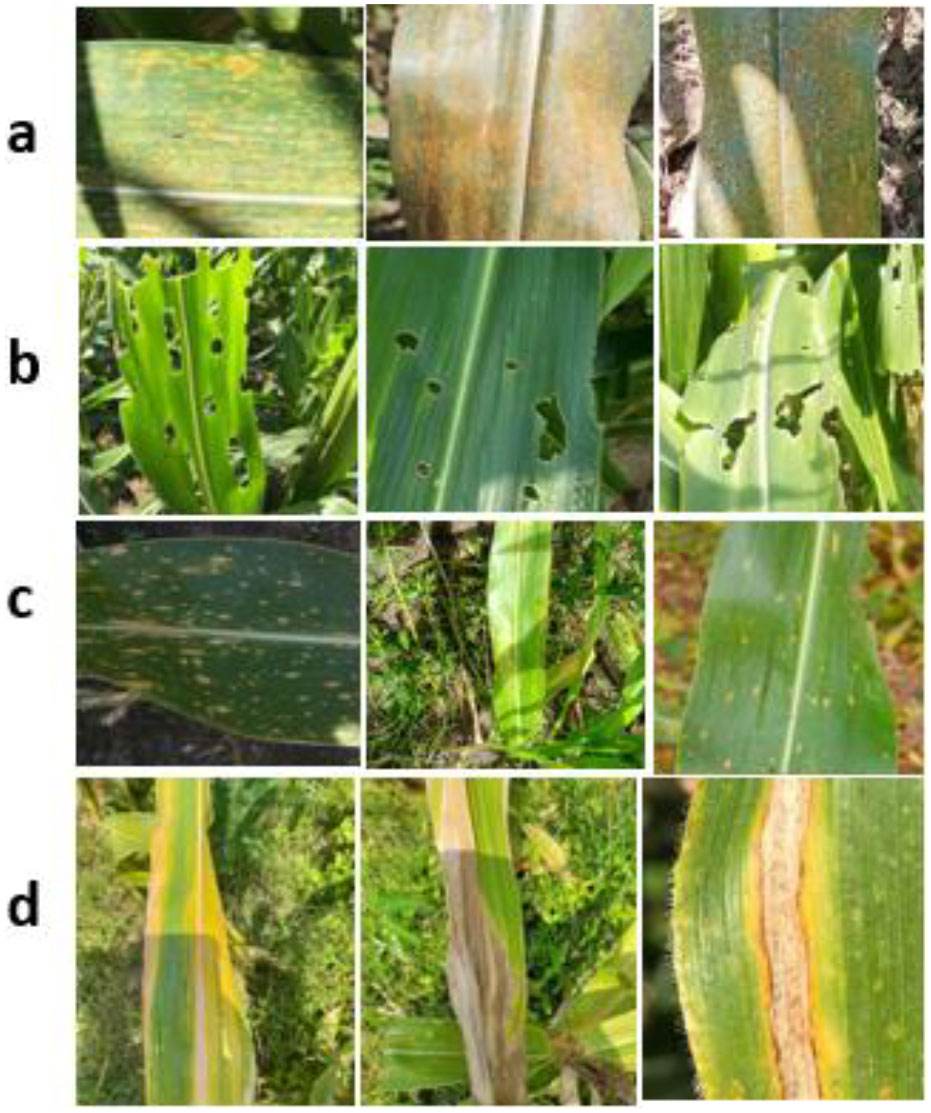
Representative samples of the collected maize field images used in this study. Each subfigure shows three sample images per category: **(a)** Common Rust, **(b)** FAW infestation, **(c)** MLB, and **(d)** TLB, illustrating the visual variability under natural field conditions.

The dataset used in this study comprises a total of 10,343 original images acquired under natural field conditions from the research farms of three institutions located in different agro-climatic zones of India, namely the Maharana Pratap University of Agriculture and Technology (MPUAT), Udaipur, the Indian Council of Agricultural Research (ICAR)–Indian Agricultural Research Institute (ICAR-IARI), New Delhi, and the ICAR–Vivekananda Parvatiya Krishi Anusandhan Sansthan (ICAR-VPKAS), Almora. These locations represent diverse climatic conditions, including arid–semiarid plains, subtropical regions, and temperate hill ecosystems, respectively. The images capture substantial variability in illumination, background complexity, leaf orientation, and disease severity, thereby reflecting realistic agricultural environments (**Figure 1**). The class-wise distribution of images is presented in **Table 1**.

**Table 1:** Class-wise distribution of the dataset.

Class	Disease of pest category	Number of images
1	MLB	2512
2	TLB	2432
3	Common Rust	2745
4	FAW	2654

The dataset was initially divided into training, validation, and testing subsets following a 60 percent, 20 percent, and 20 percent split, respectively. The partitioning was conducted in a class-balanced manner to ensure equitable representation of all disease and pest categories across each subset, thereby enabling robust training and unbiased performance evaluation. To enhance intra-class variability and reduce overfitting, image augmentation was implemented on the training dataset using custom Python scripts, with each image augmented six times by applying random horizontal flipping, rotation, scaling, brightness adjustment, contrast variation, and color jittering. This approach increased the diversity of the training samples while maintaining the independence of the validation and testing datasets for reliable model assessment.

### 2.3 Data annotation and preprocessing

Images corresponding to MLB and TLB were annotated using LabelImg, an open-source graphical image annotation tool widely used for object detection tasks. Bounding boxes were manually drawn to delineate visible disease lesions on leaf surfaces. The annotation process was carried out by trained researchers familiar with maize disease symptoms. To ensure annotation consistency, the annotated images were carefully reviewed to verify that bounding boxes accurately enclosed the visible lesion regions and followed a uniform annotation approach across the dataset.

**Figure 2:**
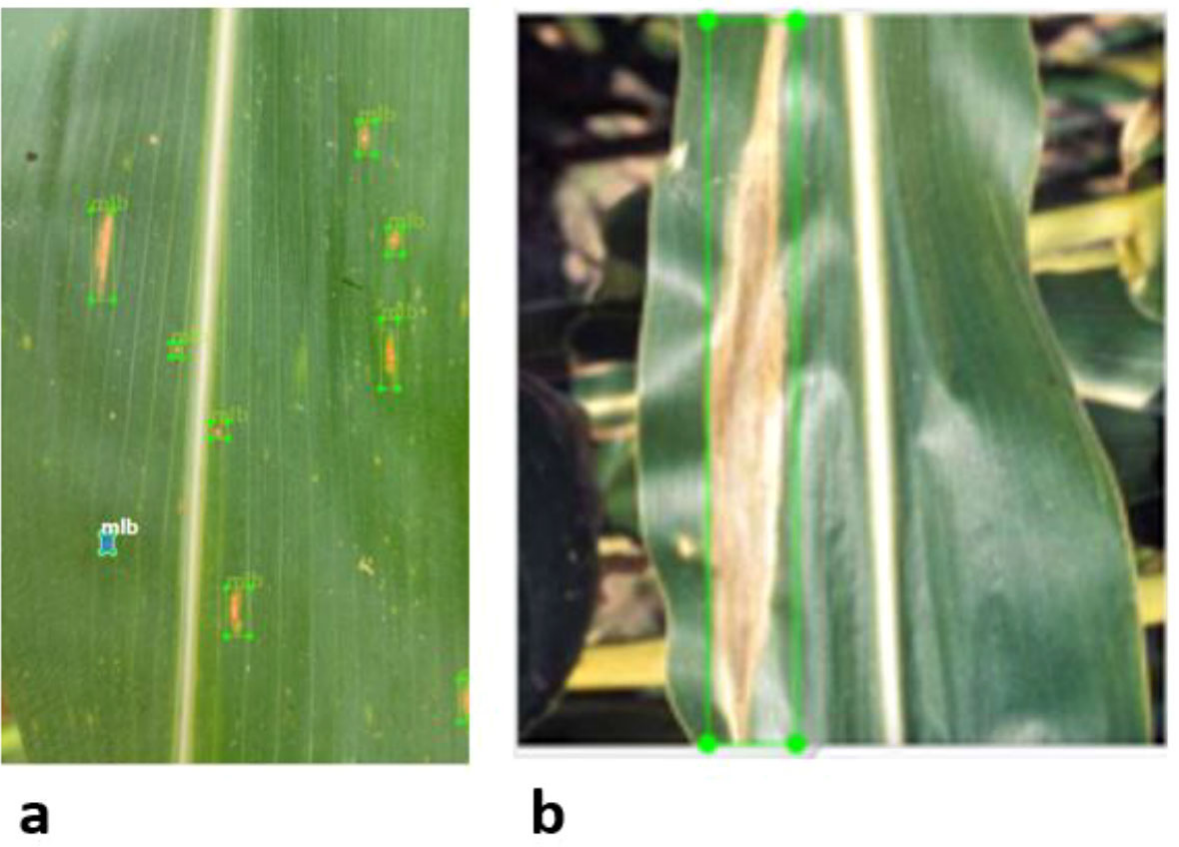
Sample annotated maize leaf images generated using *LabelImg*, illustrating bounding box annotations in YOLO format for **(a)** MLB and **(b)** TLB.

Sample annotated images illustrating bounding box placement for MLB and TLB lesions are presented in **Figure 2**, providing a visual reference for the annotation quality and lesion characteristics. All annotations were saved in the YOLO format, where each object instance is represented by its class label and normalised bounding box coordinates, including the centre position, width, and height relative to the image dimensions. In the detection model, MLB lesions and TLB lesions were annotated as separate classes, enabling the model to localise lesion regions while distinguishing between the two disease categories.

### 2.4 Classification models for common rust and FAW

#### 2.4.1 EfficientNetB3

EfficientNetB3 is a convolutional neural network based on compound scaling, which uniformly scales network depth, width, and resolution (Tan and Le, 2019). This approach enables high classification accuracy with reduced computational cost. EfficientNetB3 was selected for its ability to capture subtle texture and colour variations characteristic of Common Rust and FAW symptoms.

#### 2.4.2 MobileNetV4

MobileNetV4 is a lightweight convolutional architecture optimised for efficiency and low latency inference (Qin et al., 2025). Its design is particularly suitable for resource-constrained environments, such as mobile or edge-based agricultural monitoring systems. The model was included to evaluate the trade-off between computational efficiency and classification accuracy.

#### 2.4.3 MobileViT

MobileViT is a hybrid architecture combining convolutional layers with Vision Transformer blocks (Mehta and Rastegari, 2021). This integration allows the model to capture both local spatial features and global contextual relationships. Such capability is advantageous for distinguishing visually similar disease and pest patterns. All classification models were fine-tuned using ImageNet pretrained weights.

#### 2.4.4 Training configuration of classification models

The image classification models were trained under identical experimental conditions to ensure a fair comparison. **Table 2** summarises the hyperparameters used for training MobileNetV4, EfficientNetB3, and MobileViT.

**Table 2:** Hyperparameters used for training the image classification models.

Hyper-parameter	Value
Input image size	224 × 224
Batch size	32
Number of epochs	100
Learning rate	1 × 10^-4^
Optimiser	Adam
Loss function	Categorical cross-entropy
Weight initialisation	ImageNet pretrained weights

### 2.5 Object detection models for MLB and TLB

#### 2.5.1 YOLOv5n

YOLOv5n is a single-stage object detection framework that integrates a Cross Stage Partial backbone with a Path Aggregation Network for multi-scale feature fusion (Jocher, 2020). Its architecture enables efficient localisation and classification in real-time applications. YOLOv5n served as a baseline detector in this study due to its proven effectiveness in agricultural image analysis.

#### 2.5.2 YOLOv8n

YOLOv8n introduces an anchor-free detection head and an improved feature extraction strategy, enhancing detection accuracy for small and irregular objects (Ultralytics, 2023). These architectural refinements are particularly beneficial for leaf disease lesions, which often occupy limited spatial regions.

#### 2.5.3 YOLOv11n

YOLOv11n represents a further evolution of the YOLO family, offering improved training stability, enhanced small object sensitivity, and greater inference efficiency (Ultralytics, 2024). Its design is well-suited for detecting dense and fine-grained disease patterns present in MLB and TLB infections.

All detection models were initialised using pretrained weights and fine-tuned on the same dataset using identical training configurations to ensure a fair comparison. The key training hyperparameters used for all the YOLO models are summarised in **Table 3**.

**Table 3:** Training hyperparameters used for YOLO-based object detection models.

Parameter	Value
YOLO versions	YOLOv5n, YOLOv8n, YOLOv11n
Input image size	640 × 640
Batch size	32
Epochs	100
Learning rate	1 × 10^-^^4^
Optimiser	Adam
IoU threshold	0.5
Confidence threshold	0.25

### 2.6 Loss functions

Separate loss functions were used for object detection and image classification tasks. For MLB and TLB detection, the YOLO based models optimise a composite loss function consisting of bounding box regression loss, objectness loss, and classification loss, expressed as in Equation (1)

(1) $$L_{det}=L_{box}+L_{obj}+L_{cls}$$

where $L_{box}$ minimises localisation error using an Intersection over Union-based formulation, $L_{obj}$ measures confidence in object presence, and $L_{cls}$ penalises incorrect class predictions.

For Common Rust and FAW classification, categorical cross-entropy loss was used, and is defined as Equation (2)

(2) $$L_{cls}=-\;\sum_{i=1}^{C}y_{i}\log({\hat{y}}_{i})$$

where C denotes the number of classes (Common Rust and FAW), $y_{i}$ represent the ground truth label, and ${\hat{y}}_{i}$ denotes the predicted probability.

### 2.7 Evaluation metrics

The performance of object detection models was evaluated using precision, recall, F1 score, and mean Average Precision. Precision is defined in Equations (3–6)

(3) $$Precision=\frac{TP}{TP+FP}$$

Recall is defined as

(4) $$Recall=\frac{TP}{TP+FN}$$

The F1 Score is calculated as

(5) $$F1=\frac{2\times Precison\;\times Recall}{Precision+Recall}$$

Mean Average Precision (mAP) was computed as

(6) $$mAP=\frac{1}{N}\;\sum_{i=1}^{N}AP_{i}$$

Where AP_i_ denotes the average precision of class i, and N denotes the total number of classes, which in this study is two (MLB and TLB).

For image classification, model performance was assessed using accuracy, precision, recall, and F1 score. Classification accuracy is defined in Equation (7);

(7) $$Accuracy=\frac{TP+TN}{TP+TN+FP+FN}$$

These metrics collectively provide a comprehensive assessment of model reliability and discriminative capability.

### 2.8 System configuration and computational environment

All model development experiments were performed on an HP Z4 G5 workstation equipped with an Intel Xeon processor, 64 GB RAM, and an NVIDIA T1000 GPU with 8 GB of dedicated VRAM. This configuration provided sufficient computational resources for training and evaluating both object detection and image classification models. Model training and inference were performed using GPU acceleration to reduce computational time and ensure efficient handling of large-scale augmented datasets. The NVIDIA T1000 GPU was utilised for parallel processing during forward and backward propagation, while the CPU and system memory supported data loading, preprocessing, and augmentation operations. The hardware configuration was kept consistent across all experiments to ensure fair comparison among models and reproducibility of results.

### 2.9 Mobile application and deployment architecture

To facilitate real-world applicability and user accessibility, a mobile-based disease and pest detection system was developed. The mobile application was built using the Android platform with Software Development Kit (SDK) version 34, ensuring compatibility with recent Android operating systems and devices. The trained deep learning models were deployed using a Flask-based Application Programming Interface (API), which served as the backend inference engine. The mobile application captures or uploads maize leaf images and transmits them to the Flask API via HTTP requests. Upon receiving an input image from the mobile application, Google ML Kit is first used to filter out irrelevant images, allowing only those containing plant or leaf regions to be forwarded to the backend server. The backend then performs object detection to identify and localise maize pests or disease symptoms. When no target objects are detected, an image classification model is applied to categorise the image. This two-stage inference strategy is implemented within a client–server architecture, in which the mobile interface acts as a lightweight client and computationally intensive inference tasks are executed on the backend server. This design supports efficient mobile deployment, reduced on-device resource usage, and scalability for future large-scale agricultural monitoring applications.

## 3 Results

### 3.1 Image classification for common rust and FAW

The performance of the image classification models was evaluated for the identification of Common Rust and FAW using quantitative metrics and visual analyses. The results include training and validation behaviour, test set performance, confusion matrix analysis, and Grad-CAM–based interpretability assessment.

#### 3.1.1 Training and validation performance

**Figure 3:**
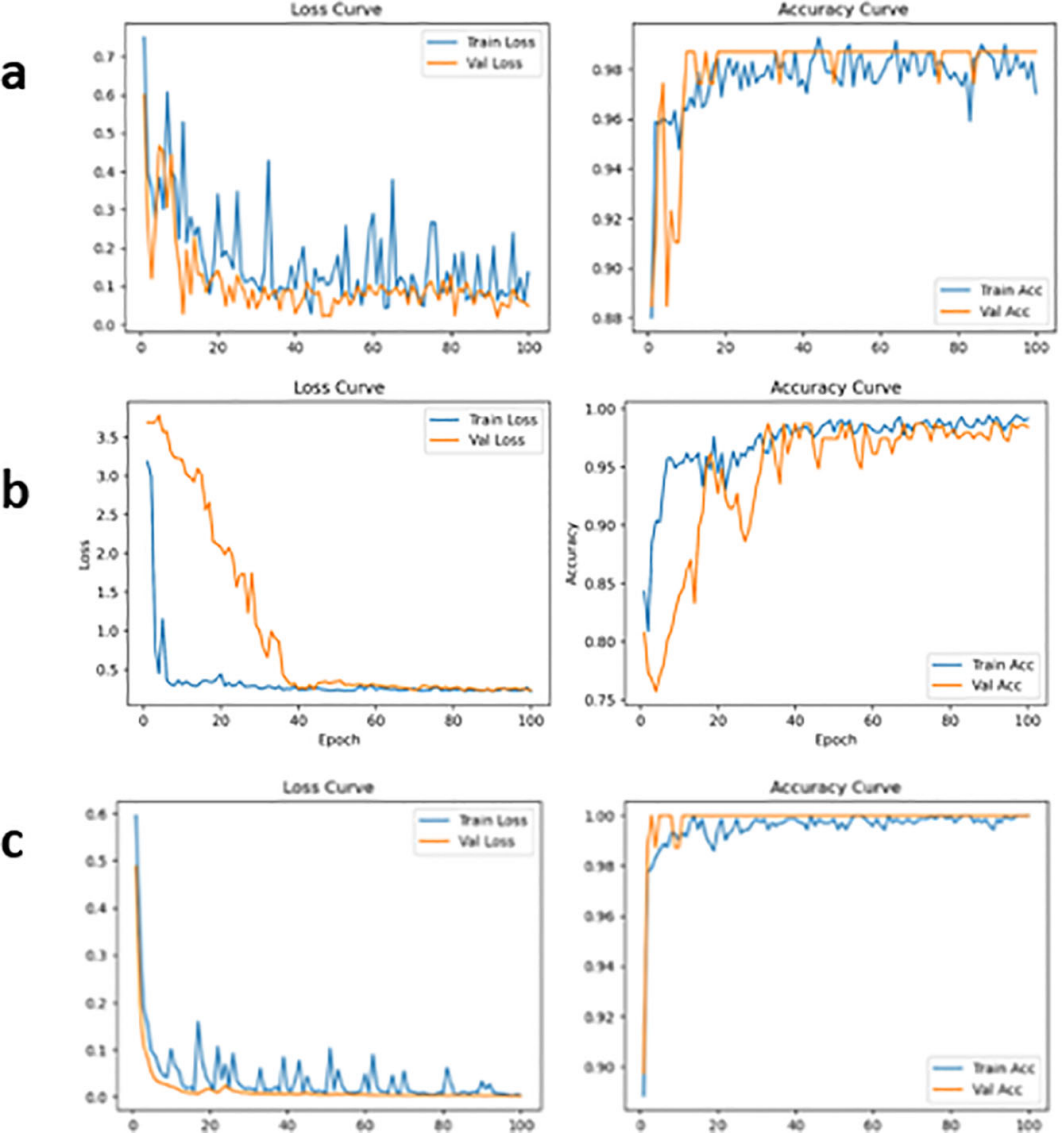
Training and validation loss and accuracy curves for the image classification models: **(a)** EfficientNetB3, **(b)** MobileNetV4, and **(c)** MobileViT.

The training and validation accuracy and loss curves for all three classification models are shown in **Figure 3**. All models exhibited stable convergence behaviour, with validation accuracy closely following training accuracy throughout the training process. The corresponding loss curves decreased steadily and stabilised at low values, indicating effective optimisation and good generalisation performance.

MobileViT and EfficientNetB3 converged more rapidly and achieved lower final loss values compared with MobileNetV4, reflecting their stronger feature representation capability. Nevertheless, MobileNetV4 maintained consistent validation accuracy across epochs, demonstrating reliable learning behaviour despite its lightweight design.

#### 3.1.2 Test set performance of image-based classification

The final evaluation was conducted on an independent test set, and the quantitative performance metrics are presented in **Table 4**. MobileViT achieved the highest classification accuracy of 99%, with corresponding precision, recall, and F1-score values of 0.989, 0.991, and 0.99, respectively. EfficientNetB3 followed with an accuracy of 97.8%, while MobileNetV4 achieved an accuracy of 96.7%, maintaining balanced precision and recall values.

**Table 4:** Test set performance of image classification models.

Model	Accuracy	Precision	Recall	F1-score
MobileNetV4	0.967	0.964	0.971	0.967
EfficientNetB3	0.978	0.976	0.980	0.978
MobileViT	0.990	0.989	0.991	0.990

These results indicate that all evaluated architectures effectively discriminate between Common Rust and FAW images collected under natural field conditions. The superior performance of MobileViT highlights the advantage of combining convolutional feature extraction with transformer-based global context modelling, while the performance of MobileNetV4 demonstrates its suitability for efficient classification in resource-constrained environments.

#### 3.1.3 Confusion matrix analysis

**Figure 4:**
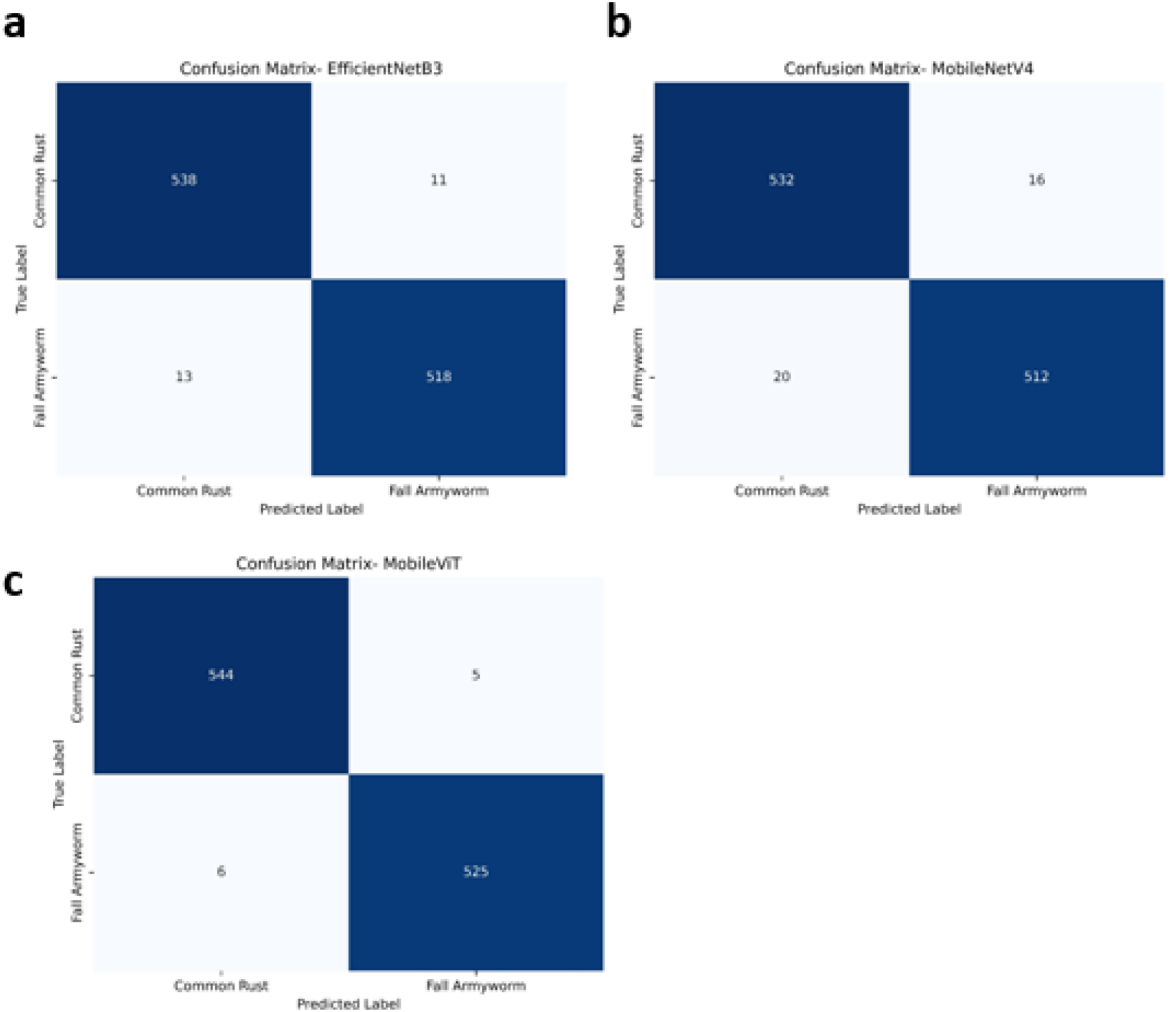
Confusion matrices illustrating class-wise prediction performance for **(a)** EfficientNetB3, **(b)** MobileNetV4, and **(c)** MobileViT.

Confusion matrix analysis further illustrates class-wise prediction behaviour (**Figure 4**). EfficientNetB3 correctly classified the majority of test samples, with limited confusion between Common Rust and FAW, as reflected by low off-diagonal values. MobileNetV4 exhibited a slightly higher number of misclassifications, particularly for visually ambiguous samples affected by background complexity or early-stage infestations.

MobileViT demonstrated the lowest misclassification rates among all models, correctly identifying nearly all samples from both classes. The reduced confusion between categories indicates strong class separability and robustness to variations in illumination, leaf orientation, and symptom severity, confirming the reliability of MobileViT for real-world maize disease and pest classification.

#### 3.1.4 Grad-CAM-based visual interpretation of classification models

**Figure 5:**
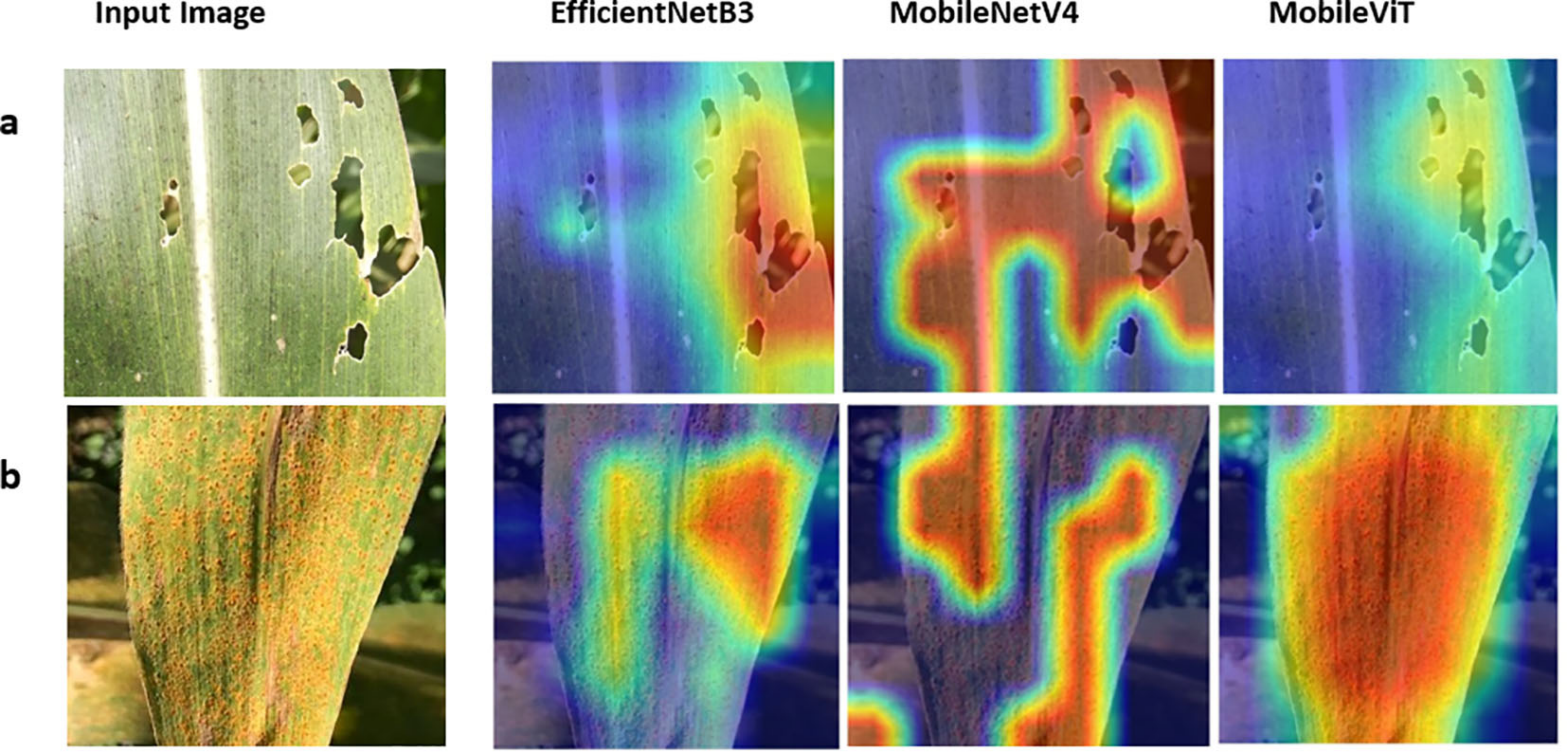
Grad-CAM visualizations for maize leaf images with a complex field background showing **(a)** Common Rust and **(b)** FAW damage, generated using EfficientNetB3, MobileNetV4, and MobileViT. Among the compared models, MobileViT demonstrates more precise and spatially consistent attention, effectively focusing on affected regions while minimizing activation in background areas.

To enhance the interpretability of the proposed classification framework, Gradient-weighted Class Activation Mapping (Grad-CAM) was applied to visualise the regions of the input images that contributed most strongly to the model predictions. Grad-CAM heatmaps were generated for representative test images of Common Rust and FAW using MobileNetV4, EfficientNetB3, and MobileViT (**Figure 5**). The Grad-CAM visualisations reveal that all three models predominantly focused on disease- or pest-affected regions of the maize leaves. For Common Rust, the activation maps highlighted characteristic rust pustules and discoloured regions, whereas for FAW images, the models concentrated on damaged leaf margins and feeding patterns. These observations indicate that the models captured biologically meaningful visual cues rather than relying on background artefacts.

**Figure 6:**
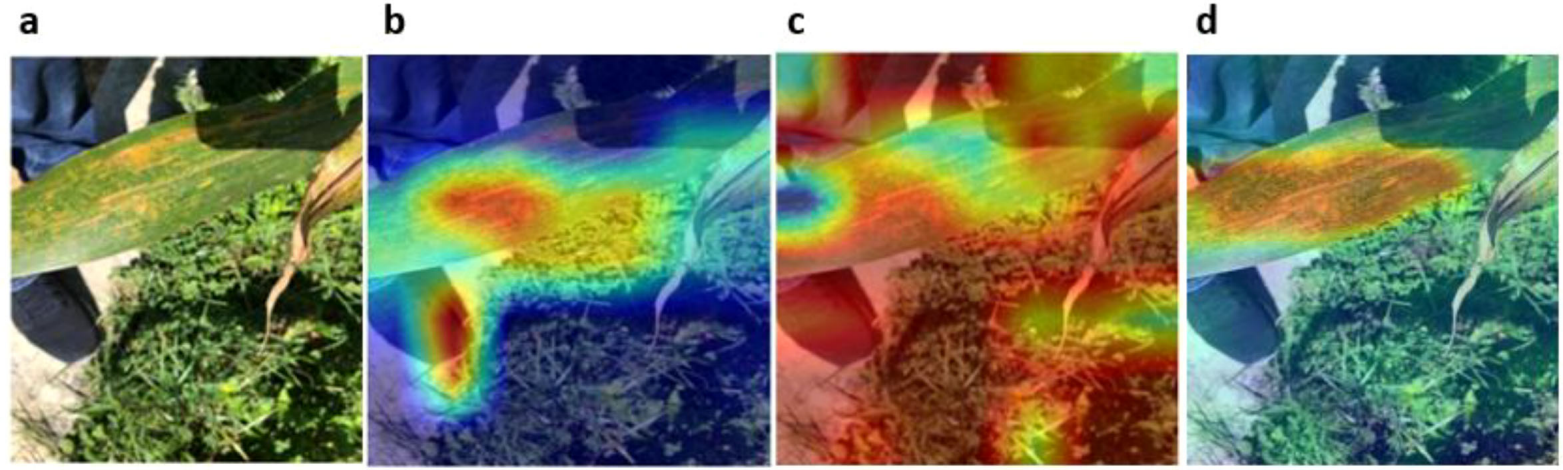
Grad-CAM visualisation of maize disease classification results showing model attention maps for a representative Common Rust sample: **(a)** original input image, **(b)** EfficientNetB3, **(c)** MobileNetV4, and **(d)** MobileViT.

Among the evaluated architectures, MobileViT exhibited the most precise and spatially consistent activation patterns, with attention tightly localised around symptomatic regions. While **Figure 5** presents representative Grad-CAM activation maps highlighting how the models generally focus on disease- or pest-affected regions, **Figure 6** illustrates a more challenging case involving complex backgrounds. In test images containing complex backgrounds with neighbouring plants and non-target foliage (**Figure 6a**), EfficientNetB3 and MobileNetV4 occasionally showed increased activation in both disease-affected regions and surrounding background areas (**Figures 6b, c**). In contrast, MobileViT focused almost exclusively on the rust-infected areas of the maize leaf, effectively suppressing background interference (**Figure 6d**). This focused attention, combined with high training, validation, and testing accuracy, indicates that MobileViT learned robust and discriminative disease-relevant feature representations. Consequently, MobileViT emerged as the most reliable model for maize disease classification among the evaluated architectures.

### 3.2 YOLO-based object detection results for MLB and TLB

YOLO-based object detection models were applied to detect MLB and TLB lesions in maize leaf images. The detection performance of YOLOv5n, YOLOv8n, and YOLOv11n was analysed using images acquired under natural field conditions characterised by complex backgrounds and variable symptom severity.

#### 3.2.1 Training performance and convergence analysis

**Figure 7:**
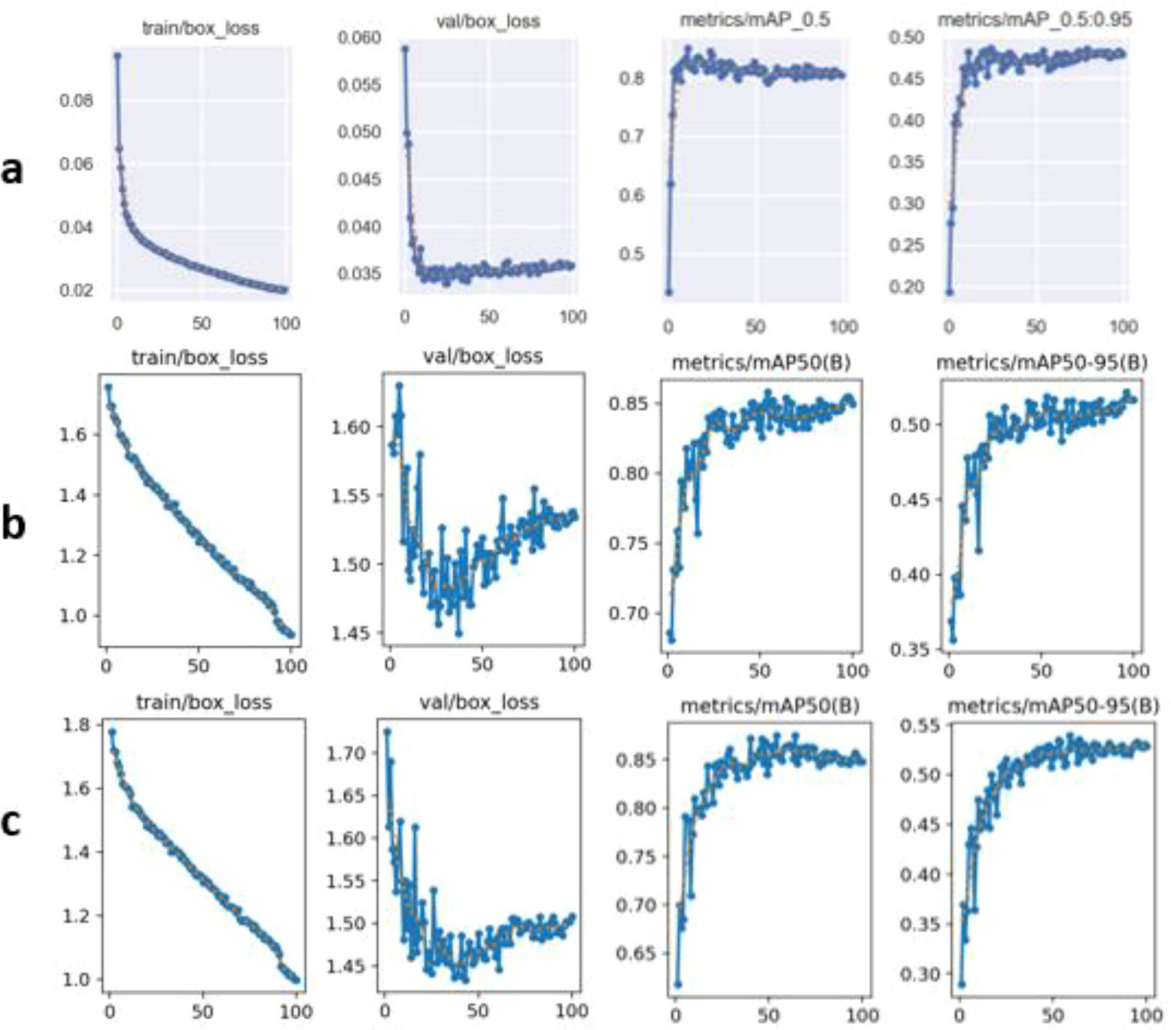
Training behaviour and convergence analysis of YOLO-based object detection models for maize leaf blight detection: **(a)** YOLOv5n, **(b)** YOLOv8n, and **(c)** YOLOv11n. For each model, the plots illustrate training and validation bounding box loss together with mAP@0.5 and mAP@0.5:0.95, highlighting differences in convergence stability and detection performance across architectures.

The training performance of the YOLO-based detection models is shown in **Figure 7**. All models exhibited a progressive reduction in training and validation bounding box loss, indicating effective learning of disease lesion localisation. However, differences in convergence stability and detection trends were observed across architectures.

YOLOv11n demonstrated the most stable convergence behaviour, with smoother loss curves and reduced validation fluctuations compared with YOLOv5n and YOLOv8n. The mAP curves further indicate that YOLOv11n consistently achieved higher mAP@0.5 and mAP@0.5:0.95 values throughout training, reflecting improved localisation accuracy for both MLB and TLB lesions. YOLOv8n showed moderate convergence stability, while YOLOv5n converged more slowly and achieved lower mAP trends.

#### 3.2.2 Quantitative evaluation on the test set

The final detection performance was evaluated on an independent test set using precision, recall, F1-score, mAP@0.5, and mAP@0.5:0.95. The quantitative results are summarised in **Table 5**. YOLOv5n achieved a precision of 0.843, a recall of 0.81, and an F1-score of 0.826, with mAP@0.5 and mAP@0.5:0.95 values of 0.850 and 0.486, respectively. YOLOv8n improved detection performance, attaining a precision of 0.858, a recall of 0.822, and an F1-score of 0.840, along with mAP@0.5 and mAP@0.5:0.95 values of 0.858 and 0.522.

**Table 5:** Test-set performance of YOLO-based object detection models for maize leaf blight detection.

Model	Precision	Recall	F1-Score	mAP@0.5	mAP@0.5:0.95
YOLOv5n	0.843	0.810	0.826	0.850	0.486
YOLOv8n	0.858	0.822	0.840	0.858	0.522
YOLOv11n	0.861	0.847	0.854	0.875	0.539

**Figure 8:**
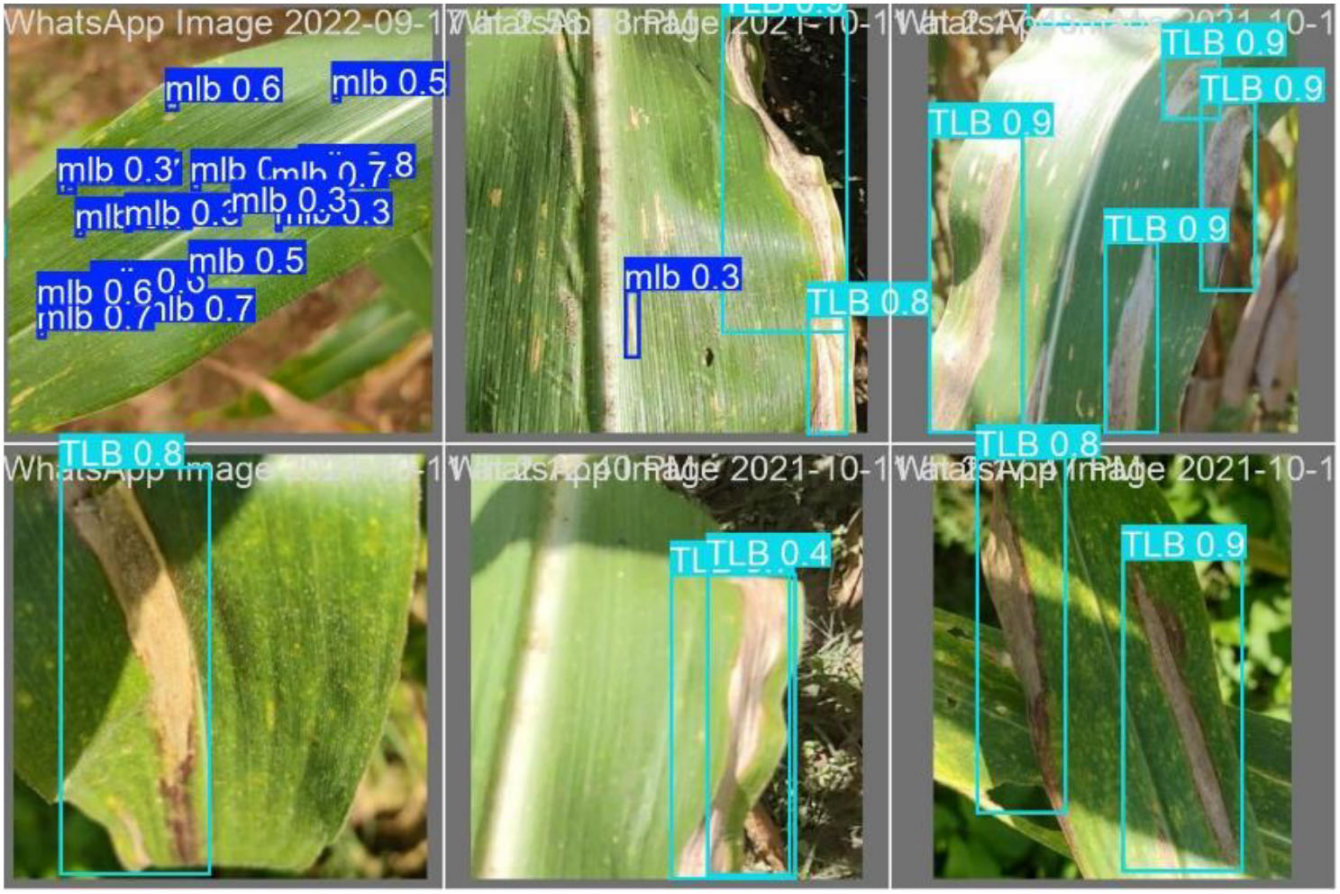
Object detection results generated by the YOLOv11n model for MLB detection. Bounding boxes and confidence scores illustrate accurate localisation of TLB and MLB lesions under natural field conditions.

Among the evaluated models, YOLOv11n achieved the highest overall detection performance, recording a precision of 0.861, a recall of 0.847, and an F1-score of 0.854. YOLOv11n also attained the highest localisation accuracy, with mAP@0.5 of 0.875 and mAP@0.5:0.95 of 0.539, indicating improved robustness across varying intersection-over-union thresholds. These quantitative improvements are further supported by the detection results shown in **Figure 8**, which illustrates representative maize leaf images with predicted bounding boxes and corresponding confidence scores. The bounding boxes indicate the spatial extent of detected lesions, while the associated confidence scores reflect the model’s certainty for each detection. YOLOv11n accurately detects both elongated TLB lesions and smaller, scattered MLB lesions, even under complex field conditions characterised by background clutter and variable symptom expression. The consistency between the high-confidence detections and the ground-truth lesion patterns visually corroborates the quantitative improvements observed in the test metrics. Together, the numerical and visual results confirm the enhanced sensitivity and reliability of YOLOv11n for maize leaf blight detection.

### 3.3 Mobile application deployment and inference results

**Figure 9:**
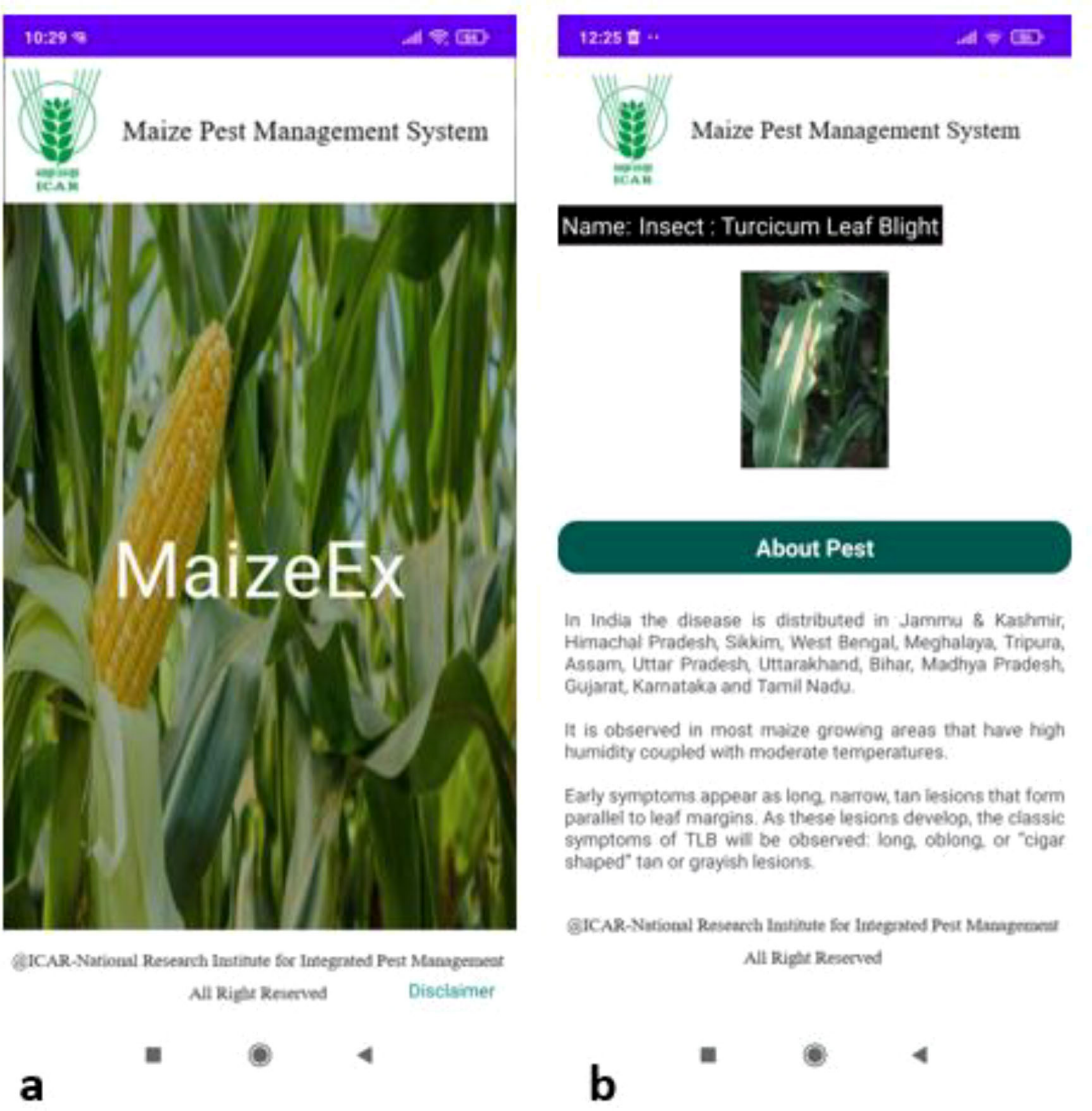
Mobile application interface and inference results for maize disease identification. **(a)** Home screen of the developed mobile application showing a simple, farmer-friendly user interface for image capture or upload. **(b)** Example inference result for TLB, where the maize leaf image is correctly classified and accompanied by corresponding management recommendations, demonstrating the application’s decision-support capability under field conditions.

To assess the field-level applicability of the proposed framework, a mobile application, named MaizeEx, was developed for on-site maize disease and pest identification. The application allows users to either capture images in real-time using the device camera or upload images from the gallery for analysis. The home interface of the application, shown in **Figure 9a**, was designed to be simple and intuitive, ensuring ease of use for farmers and extension workers.

Google ML Kit is first applied to filter out irrelevant inputs. Images that do not correspond to maize leaves or plant parts are automatically rejected, preventing erroneous inference and reducing unnecessary computational overhead.

For valid inputs, disease and pest recognition is performed using the best-performing models identified in this study, MobileViT for image-level classification and YOLOv11n for lesion-level object detection. A confidence threshold of 0.6 is applied for lesion detection using YOLOv11n, while a threshold of 0.8 is used for classification between FAW and Common Rust using MobileViT. These thresholds were selected to ensure reliable predictions and minimise false positive detections during field inference. Since the classification task does not include a healthy leaf category, the slightly higher threshold for MobileViT helps improve prediction confidence and reduces the likelihood of incorrect disease or pest identification. **Figure 9b** illustrates an example of TLB detection, where the application correctly detects the disease and provides corresponding management recommendations. This highlights the system’s ability to deliver not only accurate predictions but also actionable decision-support information.

After the disease or pest is identified from the input image, the application provides corresponding advisory information to assist users in decision-making. The management recommendations provided by the application are derived from guidelines issued by State Agricultural Universities and the Central Insecticides Board and Registration Committee (CIBRC), India, ensuring that the advisory information aligns with established agronomic practices. The mobile inference results demonstrate the feasibility of deploying the proposed deep learning framework in real-world agricultural environments, effectively bridging the gap between model development and practical, field-level disease and pest management.

## 4 Discussion

This study demonstrates the effectiveness of a unified deep learning framework for maize disease and pest analysis under natural field conditions by integrating image classification and object detection within a mobile-assisted system. The classification results indicate that all evaluated models achieved strong performance; however, the hybrid MobileViT architecture consistently outperformed purely convolutional networks. Previous studies on maize disease classification have largely relied on conventional convolutional neural networks such as VGG, ResNet, and earlier MobileNet variants (Mohanty et al., 2016; Olayiwola and Adejoju, 2023; O’Halloran et al., 2024), often trained on datasets collected by other studies or derived from public repositories (Kilaru and Raju, 2022; Nagaraju and Chawla, 2022). While such datasets have played an important role in advancing methodological research, they are commonly acquired under controlled conditions and may not fully capture the variability present in real agricultural environments.

A key strength of the present study lies in the use of a self-collected dataset acquired from research farms located in different agro-climatic zones. This multi-location data collection strategy captures substantial variability in environmental conditions, crop growth patterns, and symptom expression, which is critical for developing robust and generalisable models for field deployment. Training and evaluation using images collected across diverse climatic zones enhances the ability of the proposed framework to perform reliably under heterogeneous real-world conditions, an aspect that is often underrepresented in existing maize disease and pest detection studies.

In this context, the superior performance of MobileViT observed in this study suggests that incorporating transformer-based global context modelling enhances robustness when applied to complex field scenarios characterised by heterogeneous backgrounds and variable illumination. The ability of MobileViT to jointly capture local texture features and broader contextual relationships appears particularly beneficial for distinguishing disease symptoms from surrounding foliage. Similar advantages of hybrid convolution–transformer architectures have been reported in recent plant disease recognition studies, where improved generalisation under unconstrained field conditions was observed (Mehta and Rastegari, 2021; Yang et al., 2023). In addition, the competitive performance of MobileNetV4 supports recent findings that modern lightweight architectures can achieve high classification accuracy while maintaining computational efficiency, making them well-suited for mobile and edge-based agricultural applications (Elfatimi et al., 2022; Qin et al., 2025).

The Grad-CAM–based interpretability analysis provides further insight into model behaviour and strengthens the interpretation of the classification results. Explainability has become an important consideration in agricultural artificial intelligence, particularly for farmer-facing decision-support systems where trust and transparency are essential (Barbedo, 2018; Raveenthini et al., 2025). While earlier studies have reported that convolutional models may partially attend to background regions in complex field images, potentially leading to spurious correlations (Picon et al., 2019), the MobileViT model in this study demonstrated tightly localised attention over disease-affected regions, even in visually cluttered scenes. This focused attention on biologically meaningful symptoms provides a plausible explanation for the higher classification accuracy achieved by MobileViT and aligns with recent interpretability-driven analyses in plant pathology (Selvaraju et al., 2016; Too et al., 2019).

For lesion-level analysis, the YOLO-based object detection results reveal clear performance gains with newer detection architectures. Early applications of single-stage object detectors in plant disease and pest detection demonstrated the feasibility of localising symptomatic regions in field-acquired images using YOLO-based models (Fuentes et al., 2017; Too et al., 2019). However, accurately detecting fine-grained, irregular, and spatially dispersed disease symptoms under complex field backgrounds remains a challenging problem in agricultural computer vision. In this study, YOLOv11n achieved higher recall and mAP, indicating improved sensitivity and localisation accuracy for both MLB and TLB. These improvements are consistent with recent advances in YOLO architectures that emphasise enhanced feature fusion strategies and improved small-object detection capability (Ultralytics, 2023, 2024; Eliwa and Abd El-Hafeez, 2025).

Unlike many existing approaches that focus exclusively on either classification or detection, the proposed framework integrates both tasks within a single system and demonstrates successful deployment through a mobile application. By combining disease and pest recognition with an advisory-enabled interface, the system moves beyond algorithmic evaluation and provides actionable decision support for maize cultivation. This end-to-end design, supported by multi-agro-climatic field data, highlights the practical relevance of the proposed framework and its potential to support timely, accessible, and scalable maize disease and pest management under real field conditions.

While the proposed framework demonstrated strong performance under natural field conditions, it also provides clear opportunities for further extension and enhancement. The current study focuses on a selected set of major maize diseases and pests, establishing a robust foundation for expanding the framework to additional biotic stresses and other crop species. The present framework relies on RGB imagery, which is well-suited for practical and scalable deployment; future research may investigate the integration of multispectral or hyperspectral data to facilitate earlier disease detection and more accurate severity assessment. In addition, continued optimisation for fully on-device inference, along with the enhancement of advisory functionalities within the mobile application, will further improve scalability, usability, and real-world impact for agricultural decision-support systems.

## 5 Conclusion

This study presented a comprehensive deep learning–based framework for automated maize disease and pest identification under natural field conditions, integrating image classification, object detection, and mobile deployment within a unified system. By addressing both foliar diseases and insect pests using task-appropriate deep learning models, the proposed approach overcomes limitations of existing methods that treat these problems in isolation or rely on controlled datasets. The experimental results demonstrated that hybrid and lightweight architectures are well-suited for real-world agricultural applications. MobileViT achieved superior performance in classifying Common Rust and FAW, supported by Grad-CAM analysis that confirmed focused attention on disease-affected regions. For lesion-level analysis, YOLOv11n achieved the highest detection accuracy and robustness for MLB and TLB, effectively localising fine-grained and irregular disease patterns under complex field conditions. The use of a self-collected dataset ensured realistic evaluation and strengthened the generalisability of the findings. Beyond model performance, the successful integration of the proposed framework into a mobile application highlights its practical relevance. The application enables users to capture or upload images, filter invalid inputs, perform disease and pest identification, and provide advisory recommendations, thereby bridging the gap between algorithmic development and field-level decision support. Therefore, this work contributes a reproducible, deployment-oriented solution for precision maize health monitoring and demonstrates the potential of deep learning–driven systems to support sustainable pest management and reduced chemical dependence in agriculture.

## Data Availability

The raw data supporting the conclusions of this article will be made available by the authors, without undue reservation.
